# Neuromuscular mechanisms of weakness in a mouse model of chronic alcoholic myopathy

**DOI:** 10.1111/acer.14907

**Published:** 2022-08-02

**Authors:** Samantha E. Moser, Austin M. Brown, Brian C. Clark, W. David Arnold, Cory W. Baumann

**Affiliations:** ^1^ Honors Tutorial College Ohio University Athens Ohio USA; ^2^ Ohio Musculoskeletal and Neurological Institute (OMNI) Ohio University Athens Ohio USA; ^3^ Department of Biomedical Sciences Ohio University Athens Ohio USA; ^4^ Department of Neurology The Ohio State University Wexner Medical Center Columbus Ohio USA; ^5^ Department of Physical Medicine and Rehabilitation The Ohio State University Wexner Medical Center Columbus Ohio USA; ^6^ Department of Neuroscience The Ohio State University Wexner Medical Center Columbus Ohio USA; ^7^ Department of Physiology and Cell Biology The Ohio State University Wexner Medical Center Columbus Ohio USA

**Keywords:** atrophy, EtOH, force, skeletal muscle, strength

## Abstract

**Background:**

Weakness is a common clinical symptom reported in individuals with chronic alcohol use disorder. However, it remains unclear whether low strength in these individuals is directly related to excessive ethanol intake, other deleterious factors (lifestyle, environment, genetics, etc.), or a combination of both. Therefore, we examined whether (and how) ethanol reduces the muscle’s force‐producing capacity using a controlled in vivo preclinical mouse model of excessive ethanol intake.

**Methods:**

To establish whether chronic ethanol consumption causes weakness, C57BL/6 female mice consumed 20% ethanol for 40 weeks (following a 2‐week ethanol ramping period), and various measures of muscular force were quantified. Functional measures included all‐limb grip strength and in vivo contractility of the left ankle dorsiflexors and plantarflexors. Once confirmed that mice consuming ethanol were weaker than age‐matched controls, we sought to determine the potential neuromuscular mechanisms of muscle dysfunction by assessing neuromuscular excitation, muscle quantity, and muscle quality.

**Results:**

Mice consuming chronic ethanol were 13 to 16% weaker (*p* ≤ 0.016) than controls (i.e., mice consuming 100% water) with the negative impact of ethanol on voluntary grip strength (*ƞ* ^2^ = 0.603) being slightly larger than that of electrically stimulated muscle contractility (*ƞ* ^2^ = 0.482). Relative to controls, lean mass and muscle wet masses were 9 to 16% lower in ethanol‐consuming mice (*p* ≤ 0.048, *ƞ* ^2^ ≥ 0.268). No significant changes were observed between groups for indices of neuromuscular excitation at the level of the motor unit, neuromuscular junction, or plasmalemma (*p* ≥ 0.259, *ƞ* ^2^ ≤ 0.097), nor was muscle quality altered after 40 weeks of 20% ethanol consumption (*p* ≥ 0.695, *ƞ* ^2^ ≤ 0.012).

**Conclusions:**

Together, these findings establish that chronic ethanol consumption in mice induces a substantial weakness in vivo that we interpret to be primarily due to muscle atrophy (i.e., reduced muscle quantity) and possibly, to a lesser degree, loss of central neural drive.

## Graphical abstract

Mice consuming 20% EtOH in their drinking water for 40 weeks were 13–16% weaker than control mice (no EtOH). To determine physiological mechanisms that contribute to EtOH‐induced weakness, we evaluated several markers of neuromuscular excitation, muscle quantity, and muscle quality. Our results indicate weakness after chronic EtOH consumption is primarily due to reductions in muscle quantity (i.e., atrophy) and possibly, to a lesser degree, loss of central neural drive.

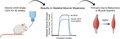

## INTRODUCTION

Excessive, long‐duration alcohol intake can lead to chronic alcoholic myopathy, a muscle disorder characterized by skeletal muscle atrophy and weakness (Fernandez‐Solà et al., 2007; Preedy et al., 2001; Urbano‐Márquez & Fernández‐Solà, 2004). Alcoholic myopathy is present in approximately 50% of chronic alcoholics (Preedy et al., 2003; Simon et al., 2017), and is most often diagnosed in middle age (Estruch et al., 1998; Martin et al., 1985; Urbano‐Márquez & Fernández‐Solà, 2004). Alcohol‐induced muscle atrophy is proportional to the total lifetime of alcohol ingestion (Lang et al., 2005; Urbano‐Marquez et al., 1989) and has been shown to lead to the erosion of up to 20% of the entire muscle mass (Lang et al., 2005; Martin et al., 1985; Urbano‐Marquez et al., 1989; Urbano‐Márquez & Fernández‐Solà, 2004). Additionally, clinically meaningful reductions in strength have been reported (Estruch et al., 1998; Urbano‐Marquez et al., 1989). For instance, chronic alcoholics (>100 g/day of EtOH) have been reported to be approximately 30% weaker than nonalcoholics (Estruch et al., 1998). Although most of these studies have been conducted in males, with little attention directed at alcohol‐induced sex differences, isolated reports suggest that females are more susceptible to alcoholic myopathy (Nemirovskaya et al., 2015; Shenkman et al., 2019). Nonetheless, in addition to being weak, chronic alcoholics also exhibit reductions in quality of life, impaired ambulation (Vancampfort et al., 2020), lower levels of physical activity (Vancampfort et al., 2020), and increased frequency of falls (Hingson & Howland, 1987).

Mechanisms that contribute to muscle weakness can include loss of neuromuscular excitation (central activation, neuromuscular junction [NMJ] transmission, and plasmalemmal excitability), muscle quantity (mass or cross‐sectional area [CSA]), and/or muscle quality (contractile efficiency or specific force) (Baumann et al., 2016; Clark & Manini, 2008). Weakness observed in chronic alcoholics has been debated to be the result of either a combination of these neuromuscular mechanisms or isolated to a single factor. For example, some have suggested that alcohol‐induced weakness is initiated by denervation‐associated peripheral neuropathy (Agelink et al., 1998; Faris & Reyes, 1971; Julian et al., 2019; Rossouw et al., 1976), while others have argued pathology originates within the muscle itself (Martin et al., 1985; Slavin et al., 1983; Trounce et al., 1987). These seemingly conflicting results may be related to difficulties quantifying and controlling for potential confounding factors such as the amount, type, and duration of alcohol intake as well as nutrition, genetics, activity levels, various socioeconomic and environmental factors (Kimball & Lang, 2018). To bypass limitations of human studies, mechanistic investigations of alcoholic myopathy have primarily been performed in preclinical rodent models by including EtOH in drinking water or as part of a nutritionally complete liquid diet.

Preclinical studies have largely focused on the molecular alterations associated with muscle atrophy rather than functional outcomes and pathophysiological mechanisms (Kimball & Lang, 2018; Lang et al., 2001; Simon et al., 2017; Slavin et al., 1983; Steiner & Lang, 2015). In fact, we are aware of only four preclinical studies over the past half century that measured the effects of excessive, long‐duration EtOH consumption on the muscle's force producing capacity (Berk et al., 1975; Crowell et al., 2019; Edmonds et al., 1987; Martyn & Munsat, 1980). Functional changes in these studies ranged from no change in the force generating capacity to ~20% deficits in force production during twitch or tetanic isometric contractions using ex vivo (also referred to as in vitro) or in situ physiology. These mixed findings could be due to various factors that include, but are not limited to, the rodent studied (rat vs. mouse), contractility parameters assessed, and the duration or concentration of EtOH used. Thus, the impact of EtOH on muscle function is not fully understood. Accordingly, the present research aimed to determine if chronic EtOH intake reduces the muscle's force producing capacity in the C57BL/6 mouse strain and, if so, to identify the putative neuromuscular site(s)/mechanisms that are responsible for EtOH‐induced weakness. These aims were accomplished by measuring in vivo muscle strength and contractility, while also comprehensively assessing indices of neuromuscular excitation, muscle quantity, and muscle quality.

## MATERIALS AND METHODS

### Ethical approval and animal models

Fifteen female C57BL/6 mice were obtained from Jackson Laboratory and aged to approximately 23 weeks prior to study initiation. Female mice were specifically selected based on human reports suggesting females are more likely to develop alcohol‐induced muscle atrophy (Nemirovskaya et al., 2015; Shenkman et al., 2019). For physiological experiments that involved anesthesia, mice were initially anesthetized in an induction chamber using isoflurane and then maintained by inhalation of 1 to 2% isoflurane mixed with oxygen at a flow rate of 200 ml/min. At the study completion, mice were again anesthetized (2 to 3% isoflurane), and the left hindlimb muscles were dissected and weighed. Mice were then euthanized by exsanguination followed by cervical dislocation in accordance with the Ohio University Animal Care and Use Committee.

### Alcohol (Ethanol (EtOH)) feeding

Ten mice were randomly assigned to receive 20% EtOH in their drinking water (EtOH group) via a no choice design, meaning the EtOH group did not have access to a 100% water bottle (Dekeyser et al., 2013; Song et al., 2002). Mice were initially acclimated to EtOH by increasing the EtOH concentration in 5% increments from 0% to the target 20% (w/v) over the course of approximately 2 weeks. The 20% concentration was continued for the next 40 weeks, for a total of 42 weeks of EtOH consumption. The target of 20% EtOH in the drinking water was selected because it has been shown to replicate blood alcohol concentrations (BACs) reported in chronic alcoholics (Collins & Neafsey, 2016; D'Souza El‐Guindy et al., 2010; Song et al., 2002) and be an appropriate laboratory model of alcohol‐induced organ damage (D'Souza El‐Guindy et al., 2010). The duration of 40 weeks at 20% EtOH was selected based on the mean lifespan of the C57BL/6 female mouse being ~120 weeks (Kwak et al., 2019), in that 40 weeks would represent drinking for 1/3 of a lifespan. We equate this to a human drinking ~25 years and living for ~75 years. Essentially, heavy EtOH consumption for a 25‐year‐old individual drinking into their late 40s. Five mice served as controls (control group) and were given 100% water over the duration of the study. Standard rodent chow was supplied ad libitum to both groups.

### Experimental design

To establish if chronic EtOH consumption causes weakness, mice consumed 20% EtOH for 40 weeks (following the 2‐week EtOH ramping period), and indices of muscular force production were measured. Functional measures included all‐limb grip strength, as well as in vivo contractility of the left ankle dorsiflexors (tibialis anterior, extensor digitorum longus, and extensor hallucis muscles) and plantarflexors (gastrocnemius, soleus, and plantaris muscles). Once confirmed that mice consuming EtOH were weaker than age‐matched controls, we sought to determine the underlying neuromuscular mechanisms. These mechanisms were categorized into three factors/sites that included neuromuscular excitation, muscle quantity, and muscle quality.

#### Neuromuscular excitation

For this study, loss of neuromuscular excitation was classified as any disruption in the ability to excite the muscle and included all processes at or above plasmalemmal activation. Central drive was assessed by comparing functional deficits obtained for conscious grip strength (i.e., voluntary) to that of anesthetized in vivo torque (i.e., nonvoluntary, electrically‐stimulated). A greater deficit in grip strength compared with in vivo torque for EtOH consuming mice, when expressed relative to control values, was interpreted as loss of central (neural) activation. Additionally, we assessed potential physiological mechanisms that relate to neuromuscular excitation. These included: (1) motor unit number and size estimates, (2) indices of NMJ transmission, and (3) plasmalemmal excitability.

#### Muscle quantity

Atrophy or loss of muscle quantity was measured using two different approaches. The first was completed in living mice using nuclear magnetic resonance (NMR) to calculate lean body mass. The second was at the time of euthanasia, at which point wet mass of the left anterior crural muscles and posterior crural muscles were obtained. Loss of lean mass or muscle wet mass was used to assess atrophy.

#### Muscle quality

Reduced muscle quality was defined as a decrease in the muscle's intrinsic force‐producing capacity and attributed to excitation–contraction coupling failure, impaired cross‐bridge cycling, and/or altered muscle composition (e.g., increased fibrosis). In general, muscle quality was analyzed by normalizing muscle function to muscle mass (i.e., specific force). Grip strength was normalized to lean mass to measure grip strength quality while tetanic isometric torque was normalized to wet muscle mass to measure quality of contractility (e.g., dorsiflexor torque relative to anterior crural muscle mass). A decrease in specific force, when compared with control values, indicated EtOH consumption caused a reduction in muscle quality. Excitation–contraction coupling failure, a specific component of muscle quality, was also indirectly assessed. In skeletal muscle, excitation–contraction coupling failure is broadly defined as any disruption in the sequence of events linking the activation of the voltage‐sensitive dihydropyridine receptors to the release of Ca^2+^ through the ryanodine receptors. To assess excitation–contraction coupling failure in the ankle dorsiflexors, low‐ and high‐frequency (i.e., twitch/tetanic) isometric torques were compared. More pronounced reductions in twitch relative to tetanic torque have traditionally been used to indicate greater excitation–contraction coupling failure (Ingalls et al., 2004; Jones et al., 1982).

### Experimental methodology

Measurements were completed within 4 weeks prior to euthanasia. All‐limb grip strength and body composition were completed on the same day. Approximately 1 week separated each measurement; hindlimb muscle isometric torque, electromyography, and all‐limb grip strength plus body composition. To note, EtOH did not impact survival nor were any obvious health complications observed in the EtOH group over the duration of this study.

#### Hindlimb muscle isometric torque

In vivo isometric torque of the left ankle dorsiflexors and plantarflexors was assessed as previously described (Baumann et al., 2014; Cabelka et al., 2019; Lowe et al., 1995). The anesthetized mouse (see section Ethical Approval and Animal Models) was placed on a temperature‐controlled platform set at approximately 38 to 40°C to maintain core temperature. The left knee was clamped, and the left foot was secured to a footplate attached to the shaft of the servomotor system (Model 300C‐LR; Aurora Scientific) with the foot perpendicular to the tibia (defined as 0°). Sterilized needle electrodes, connected to the stimulator and stimulus isolation unit (701C; Aurora Scientific), were initially inserted percutaneously on either side of the peroneal nerve that innervates the dorsiflexor muscles. Stimulation voltage and needle electrode placement were optimized with isometric tetanic contractions (200 ms train of 0.2 ms pulses at 250 Hz). Contractile function of the ankle dorsiflexor was then assessed by measuring isometric torque as a function of stimulation frequency (10 to 300 Hz); the highest recorded torque was defined as dorsiflexor tetanic isometric torque. Ankle plantarflexor torque was then performed in a similar manner with some minor modifications. Briefly, the needle electrodes were instead inserted percutaneously on either side of the sciatic nerve that branches to the tibial nerve, thus innervating the ankle plantarflexor muscles. Stimulation voltage and needle electrode placement were again optimized, and plantarflexor tetanic isometric torque was recorded (200 ms train of 0.2 ms pulses at 250 Hz).

#### All‐limb grip strength

Grip strength was evaluated using a grip meter test (Grip meter; Model GT3, Bioseb) (Baumann et al., 2018) and performed at 9:00 a.m. Mice were gently lowered over the top of a wire grid so that the front and hind paws gripped the grid. The tail of each mouse was pulled back steadily, keeping the mouse's torso in a horizontal position after the mouse had a firm grasp of the grip. When the mouse was unable to maintain its grip, the trial was over and the grip strength (grams) was recorded. Each mouse performed three trials with a 10‐min rest period in‐between each trial. The best score of these trials was used as peak grip strength.

#### Electromyography (EMG)

Compound motor action potential (CMAP) amplitudes were recorded from the left hindlimb as previously described using a clinical electrodiagnostic system (Cadwell) (Arnold et al., 2015). In anesthetized mice (see section Ethical Approval and Animal Models), two insulated 28‐gauge monopolar needle electrodes (Teca, Oxford Instruments Medical) were inserted subcutaneously in the proximal hindlimb to stimulate the sciatic nerve. Two fine wire ring electrodes (Alpine Biomed) were used as recording electrodes and were placed distal to the knee over the bulk of the gastrocnemius muscle (active recording electrode) and over the metatarsal region of the foot (reference recording electrode). A disposable ground electrode (Carefusion) was placed on the tail. To measure the CMAP, the left sciatic nerve was supramaximally‐stimulated (<10 mA constant current intensity, 0.1 ms pulse duration, 20 mV sensitivity, 10 Hz to 10 kHz filter), and the peak‐to‐peak amplitudes were recorded. Motor unit number estimate (MUNE) was determined using an incremental technique (Arnold et al., 2015). A total of 10 incremental submaximal responses (50 to 500 *μ*V sensitivity, 10 Hz to 10 kHz filter) were recorded and averaged to determine the single motor unit potential (SMUP) amplitude. MUNE was calculated as follows: MUNE = CMAP amplitude peak‐to‐peak/Average SMUP peak‐to‐peak.

Repetitive nerve stimulation (RNS) was performed to assess the ability of the NMJ to generate a muscle fiber action potential (i.e., NMJ transmission) (Padilla et al., 2021). Decrement on RNS reflects reduction of the summated excitation of a muscle following repetitive supramaximal stimulation of a peripheral nerve. During RNS, placement of electrodes for stimulation, grounding, and recording were the same as described for CMAP. Repetitive CMAP amplitudes were recorded following a train of 10 supramaximal stimuli delivered to the sciatic nerve at 50 Hz. Change of CMAP amplitude between the first stimulation and the 10th stimulation (decrement) was calculated with the following equation: % Amplitude decrement = [(Amplitude of 10th response − Amplitude 1st response)/Amplitude of 1st response] × 100%.

#### Body composition

Mice were scanned using the Bruker Minispec NMR Analyzer (LF50 Series, model mq 7.5). The NMR system acquires and analyzes signals from the sample volume in order to determine fat, free body fluid, and lean tissue values. The system was calibrated before testing using a standard provided by the manufacturer. The mice were placed into small plastic cylinder tubes with air holes, and a tight‐fitting plunger was inserted into the cylinder to immobilize the mice. This tube was then placed into the sample chamber of the analyzer for approximately 2 min or until the scan was complete.

#### Blood alcohol concentration (BAC)

Blood was collected at study completion via a cardiac puncture within 1 h after the dark cycle ended (5:00 to 7:00 a.m.). Approximately 200 to 250 *μ*l of blood was collected into an Eppendorf tube via an 18‐gauge sterile needle, stored on ice, and then centrifuged at 4°C for 5 min at 10,000 × *g*. Serum was frozen at −80°C until BAC (mg/dl) was determined using an AM1 alcohol analyzer (Analox Instruments Ltd.).

### Statistical analyses

To determine group differences between the EtOH and control groups, an unpaired *t*‐test was utilized. A *p* value of <0.05 was required for statistical significance. Eta^2^ effect sizes (*ƞ* ^2^) are reported as an additional statistical parameter to aid in interpreting the magnitude of effects in the findings. Values are presented as mean ± SD. All statistical testing was performed using GraphPad Prism 9.3.1 (GraphPad Software).

## RESULTS

### Blood alcohol concentrations are elevated in EtOH consuming mice

**FIGURE 1:**
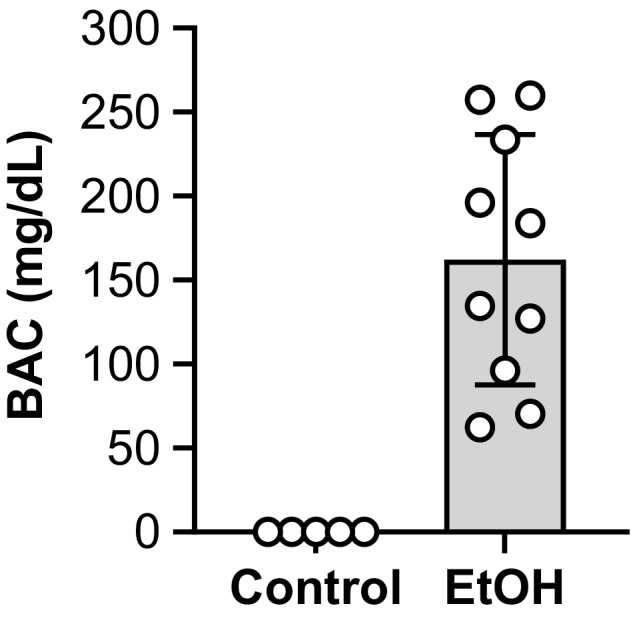
Blood alcohol concentrations (BACs) were elevated after chronic EtOH consumption. All mice were female and the EtOH group consumed 20% EtOH in drinking water for approximately 40 weeks. Blood was collected at study completion via a cardiac puncture within 1 h after the dark cycle ended (5:00 to 7:00 a.m.). bars are mean ± SD. *N* = 10 for EtOH and 5 for control. EtOH, Ethanol.

Once intake of EtOH was increased up to 20%, mice consumed approximately 21.88 g of EtOH per kg of body mass each day. Mouse fluid intake of the EtOH group was on average 2.68 ml/day compared with 3.77 ml/day for the control group (*p* < 0.001, *ƞ* ^2^ = 0.668). At study completion, the BACs of the EtOH group ranged from 62 to 260 mg/dl (Figure 1). These BACs are similar to what others reported when blood was collected after nocturnal feeding of 20% EtOH in drinking water (BACs up to 400 mg/dl) (Song et al., 2002). No detectable levels of EtOH were observed in control mice.

### Chronic EtOH intake results in skeletal muscle weakness

**FIGURE 2:**
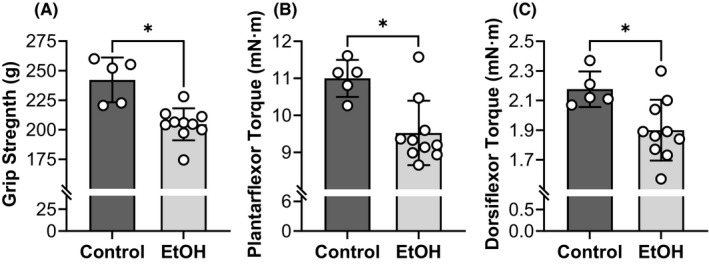
Chronic EtOH intake resulted in skeletal muscle weakness. Grip strength was evaluated by assessing voluntary all‐limb grip strength (A) while contractility of left plantarflexors (gastrocnemius, soleus, and plantaris muscles) (B) and dorsiflexors (tibialis anterior, extensor digitorum longus, and extensor hallucis muscles) (C) were measured by obtaining tetanic isometric torques from the left hindlimb. All mice were female and the EtOH group consumed 20% EtOH in drinking water for approximately 40 weeks. *Significantly different than the control group (*p* < 0.05). Bars are mean ± SD. *N* = 10 for EtOH and 5 for control. EtOH, Ethanol.

All‐limb grip strength produced by EtOH consuming mice was 16% less than that of controls (*p* < 0.001) (Figure 2A). Similarly, tetanic isometric torques of the left plantarflexors and dorsiflexors were both 13% less in the EtOH group compared with the control group (*p* ≤ 0.016) (Figure 2B,C). Deficits in grip strength did not differ from that of the left plantarflexor or dorsiflexor torques (*p* ≥ 0.413, *ƞ* ^2^ ≤ 0.076); however, the eta^2^ effect size for grip strength was the larger (*ƞ* ^2^ = 0.603) than that of plantarflexor torque (*ƞ* ^2^ = 0.482) or dorsiflexor torque (*ƞ* ^2^ = 0.369).

### No overt electromyographic changes were detected after chronic EtOH consumption

**FIGURE 3:**
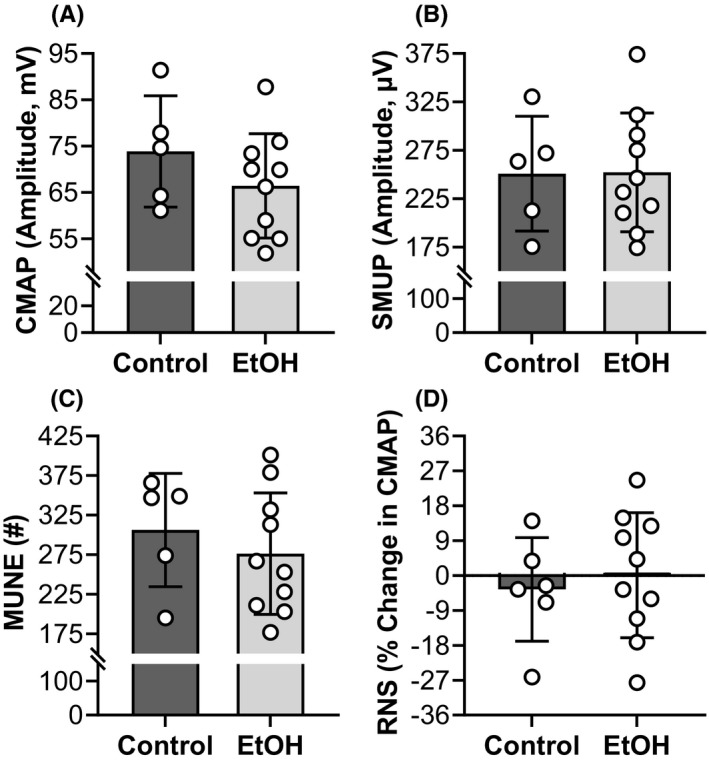
No overt electromyographic changes were detected after chronic EtOH consumption. Various in vivo parameters of NMJ transmission and plasmalemma excitability (A to D) were assessed by stimulating the sciatic nerve and measuring the electromyographic signal of the left posterior crural muscles, primarily the gastrocnemius. All mice were female and the EtOH group consumed 20% EtOH in drinking water for approximately 40 weeks. Bars are mean ± SD. *N* = 10 for EtOH and 5 for control. CMAP, compound motor action potential; EtOH, Ethanol; MUNE, motor unit number estimate; RNS, repetitive nerve stimulation; SMUP, single motor unit potential. EtOH, Ethanol.

Various in vivo parameters of NMJ transmission and plasmalemma excitability were assessed by stimulating the sciatic nerve and measuring the electromyographic signal of the posterior crural muscles, primarily the gastrocnemius. CMAP, calculated as peak‐to‐peak amplitude, did not differ between the EtOH and control groups (*p* = 0.259, *ƞ* ^2^ = 0.097) (Figure 3A). Similarly, EtOH consumption did not influence the SMUP (*p* = 0.969, *ƞ* ^2^ < 0.001) (Figure 3B). No differences were observed between the EtOH and control group for the MUNE or CMAPs in response to RNS (*p* ≥ 0.482, *ƞ* ^2^ ≤ 0.039) (Figure 3C,D).

### Body mass, body composition, and muscle wet mass are altered by chronic EtOH intake

**FIGURE 4:**
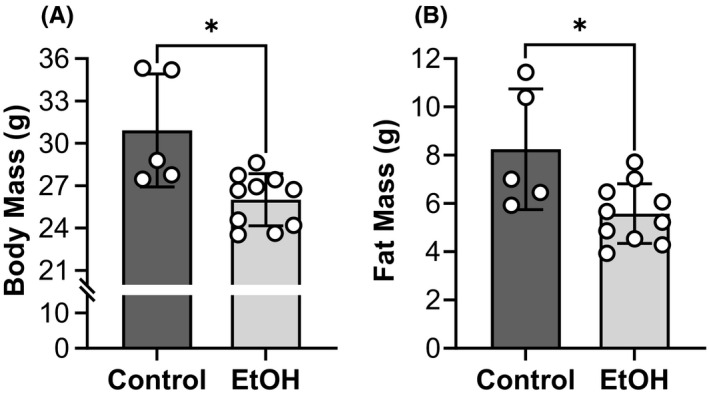
Body mass and fat mass were lower in the EtOH group. Body mass (A) was obtained used an electric balance while fat mass (B) was assessed using nuclear magnetic resonance (NMR). Lean mass is presented in Figure 5A. All mice were female and the EtOH group consumed 20% EtOH in drinking water for approximately 40 weeks. *Significantly different than the control group (*p* < 0.05). Bars are mean ± SD. *N* = 10 for EtOH and 5 for control. EtOH, Ethanol.

**FIGURE 5:**
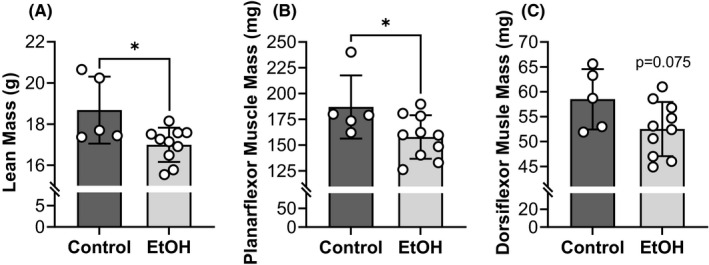
Lean mass and muscle wet mass were reduced by chronic EtOH intake. Atrophy or loss of muscle quantity was assessed using nuclear magnetic resonance (NMR) to estimate lean body mass (A) and by obtaining muscle wet mass of the left plantarflexors (gastrocnemius, soleus, and plantaris muscles) (B) and dorsiflexors (tibialis anterior, extensor digitorum longus, and extensor hallucis muscles) (C). All mice were female and the EtOH group consumed 20% EtOH in drinking water for approximately 40 weeks. *Significantly different than the control group (*p* < 0.05). Bars are mean ± SD. *N* = 10 for EtOH and 5 for control. EtOH, Ethanol.

Mouse body mass averaged 21.37 g at baseline (i.e., prior to EtOH feeding) and did not differ between mice designated for the EtOH and control groups (20.93 ± 1.58 vs. 22.24 ± 0.94 g, *p* = 0.114, *ƞ* ^2^ = 0.181). Over the course of the study, both groups gained body mass (*p* < 0.001, *ƞ* ^2^ = 0.837); however, mice consuming EtOH weighed ~16% less than the controls after EtOH feeding (*p* = 0.006, *ƞ* ^2^ = 0.458) (Figure 4A). Due to the difference in body mass between groups, body composition was analyzed using NMR. The EtOH group maintained less grams of fat (32%, *p* = 0.015, *ƞ* ^2^ = 0.379) (Figure 4B) and lean mass (9%, *p* = 0.018, *ƞ* ^2^ = 0.361) (Figure 5A) compared with the control group. When fat and lean mass were normalized to body mass (i.e., percent fat or lean mass), the EtOH group had a lower body fat percentage (21.27 ± 3.37 vs. 26.21 ± 4.60%, *p* = 0.033, *ƞ* ^2^ = 0.303) but greater percentage of lean mass (65.48 ± 2.85 vs. 60.69 ± 2.60%, *p* = 0.008, *ƞ* ^2^ = 0.433). Wet mass of the left hindlimb muscles was 14% less in the EtOH group than that of the control group (210.36 ± 22.48 vs. 245.48 ± 27.36 mg, *p* = 0.020, *ƞ* ^2^ = 0.353). When analyzed as individual muscle groups, the left posterior crural muscles weighed 16% less compared with controls (*p* = 0.048, *ƞ* ^2^ = 0.268) (Figure 5B), while the left anterior crural muscles tended to weigh 10% less (*p* = 0.075, *ƞ* ^2^ = 0.224) (Figure 5C).

### Skeletal muscle quality is maintained after chronic EtOH consumption

**FIGURE 6:**
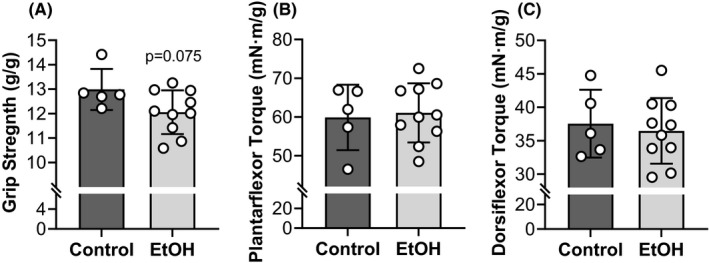
Skeletal muscle quality was maintained after chronic EtOH consumption. Muscle quality was analyzed by normalizing muscle function to lean/muscle mass (i.e., specific force). Grip strength was normalized to lean mass to measure grip strength quality (A) while tetanic isometric torque was normalized to wet muscle mass to measure quality of contractility. Specifically, plantarflexor torque was normalized to the wet mass of the posterior crural muscle (gastrocnemius, soleus, and plantaris muscles) (B) and dorsiflexor torque was normalized to the wet mass of the anterior crural muscle (tibialis anterior, extensor digitorum longus, and extensor hallucis muscles) (C). See Figure 2 for force measurements and Figure 5 for lean/muscle masses. All mice were female and the EtOH group consumed 20% EtOH in drinking water for approximately 40 weeks. Bars are mean ± SD. *N* = 10 for EtOH and 5 for control. EtOH, Ethanol.

Specific grip strength and isometric torque were determined by (1) normalizing grip strength to lean mass and (2) tetanic isometric torque to wet muscle mass, respectively. For specific torque, in vivo plantarflexor torque was normalized to the posterior crural muscles and in vivo dorsiflexor torque was normalized to the anterior crural muscles. Specific grip strength tended to be lower in the EtOH group compared with the control group, however, was not statistically significant (*p* = 0.075, *ƞ* ^2^ = 0.224) (Figure 6A). Specific torque of the plantarflexors and dorsiflexors also did not differ between groups (*p* ≥ 0.695, *ƞ* ^2^ ≤ 0.012) (Figure 6B,C).

To indirectly assess excitation–contraction coupling failure in the ankle dorsiflexors, low‐ and high‐frequency (i.e., twitch/tetanic) isometric torques were evaluated. When compared with control values, reduction in twitch isometric torque was similar to that of tetanic isometric torque (11 vs. 13%, *p* = 0.763, *ƞ* ^2^ = 0.005). Therefore, the twitch‐to‐tetanic torque ratio also did not differ between the EtOH and control groups (0.26 vs. 0.25, *p* = 0.769, *ƞ* ^2^ = 0.007).

## DISCUSSION

Weakness is a common clinical symptom reported in chronic alcoholics; yet it remains unclear if low strength in chronic alcoholics is directly associated with excessive EtOH intake, other factors (lifestyle, environment, genetics, etc.), or a combination of both. Therefore, we set out to determine if EtOH reduces the muscle's force producing capacity using a controlled in vivo preclinical mouse model of excessive EtOH intake. Moreover, if EtOH‐induced weakness was observed, we sought to examine which neuromuscular mechanisms (neuromuscular excitation, muscle quality, and/or muscle quantity) were responsible for the deleterious effects of EtOH on muscle function. Three primary findings were observed. First, mice consuming chronic EtOH (20%) were 13 to 16% weaker, as measured by voluntary grip strength and electrically‐stimulated muscle contractility, when compared with controls (i.e., mice consuming 100% water). Moreover, the eta^2^ effect size observed for the impact of EtOH on voluntary grip strength (*ƞ* ^2^ = 0.603) was slightly larger than that of electrically‐stimulated muscle contractility (*ƞ* ^2^ = 0.482). Second, relative to controls, lean mass and muscle wet masses were notably reduced 9 to 16% in EtOH consuming mice. Third, indices of neuromuscular excitation at the level of motor unit, NMJ, and plasmalemma, in addition to muscle quality, were not significantly altered after long duration, high EtOH intake. Together, these findings establish that chronic excessive EtOH consumption in mice does induce a considerable amount of weakness in vivo that we suggest is primarily due to muscle atrophy and possibly, to a lesser degree, loss of central neural drive.

In general, neuromuscular mechanisms driving weakness may include loss of neuromuscular excitation, muscle quality, and/or muscle quantity (Baumann et al., 2016; Clark & Manini, 2008). The etiology of weakness observed in chronic alcoholics has been debated to be the result of either a combination of these neuromuscular mechanisms or isolated to a single factor (Faris & Reyes, 1971; Martin et al., 1985; Rossouw et al., 1976; Slavin et al., 1983; Trounce et al., 1987). Of the few preclinical studies to measure muscle function after chronic EtOH consumption and find meaningfully deficits, Crowell et al. (2019) observed muscle‐stimulated ex vivo force of the extensor digitorum longus was reduced 20% after C57BL/6 male mice consumed a 30% EtOH containing liquid diet for 14 to 16 weeks. Moreover, suggested that EtOH‐induced loss of ex vivo muscle force was due to loss of muscle quantity rather than muscle quality (Crowell et al., 2019). However, by using ex vivo physiology, all aspects of muscular contraction upstream of the voltage‐activated ions channels within the plasmalemma are bypassed. Ex vivo muscle physiology (where only direct muscle stimulation is performed) may therefore miss possible mechanisms of dysfunction that occur due to plasmalemmal action potential propagation or any upstream process. This presents a limitation in ex vivo physiology, as alcohol‐related peripheral neuropathy is a well‐described complication of chronic alcoholism in humans (Julian et al., 2019; Mellion et al., 2011), in addition to possible perturbations at the plasmalemma that have been observed in EtOH fed rats (Adachi et al., 2003; Worrall et al., 2001). To gain a more complete understanding of neuromuscular mechanisms driving EtOH‐induced weakness, we performed a comprehensive in vivo study that assessed indices of neuromuscular excitation, muscle quality, and muscle quantity. In the following sections, we outline each of these functional sites. However, prior to our discussion, we want to be transparent that the conclusions being drawn are based on data collected at one specific timepoint, which may not capture the more acute events. We will further discuss this limitation in section entitled Future Directions.

### Neuromuscular excitation

Here, we define loss neuromuscular excitation as any disruption in the ability to excite the muscle and thus, includes processes at and/or above plasmalemma activation. The capacity of the nervous system to volitionally activate skeletal muscle was evaluated by comparing functional deficits obtained for conscious grip strength (i.e., voluntary) to that of anesthetized in vivo torque (i.e., non‐voluntary). Excessive EtOH intake exerted strong negative effects on muscle function as noted by the substantial reductions detected in both grip strength (16%) and electrically‐stimulated contractile torque (13%) when compared with values of control mice (Figure 2). Although these deficits did not differ, the observation of a more robust eta^2^ effect size for grip strength (*ƞ* ^2^ = 0.603) vs. contractile torque of the plantarflexors (*ƞ* ^2^ = 0.482) and dorsiflexors (*ƞ* ^2^ = 0.369) suggests that this study may have been underpowered or that EtOH consumption may contribute to weakness by reducing central activation. It is well‐known that alcohol results in notable alterations in the brain structure and function. With regard to the motor system, some chronic alcoholics have been reported to have prolonged central motor conduction times (Oishi & Mochizuki, 1999; Ono et al., 1999) and reduced volumes of key striatal motor control structures (Sullivan et al., 2005). Moreover, acute and chronic exposure to alcohol can modulate ion channels that regulate neuronal excitability (Harrison et al., 2017; Roberto & Varodayan, 2017). Thus, there is a strong theoretical basis for why reductions in central neural drive could be partially responsible for EtOH‐induced weakness.

Neuromuscular excitation, downstream of central nervous system activation, requires integrity of the motor axon, NMJ transmission, and plasmalemma, all of which were measured in our study via various electromyographic assessments. Specifically, excessive EtOH intake did not significantly alter indices of plasmalemmal excitability as measured by CMAP amplitude, motor axonal/unit number as measured by MUNE, or NMJ transmission as measured by RNS (Figure 3). Our results demonstrate that the ability of motor axon to excite the muscle is functionally intact, but it is important to note that we cannot rule out subtle motor axonal action potential propagation defects (conduction velocity changes) or defects of sensory axonal function. Prevalence of mixed sensory and motor axonal peripheral neuropathy has been reported to be ~45% in chronic alcohol abusers and is thought to occur in the absence of nutritional deficiency (Julian et al., 2019; Mellion et al., 2011). Moreover, conduction velocities of large myelinated fibers of the sciatic nerve were observed to be slower in rat feds EtOH for 7+ months (Juntunen et al., 1978), while others have reported EtOH may lead to membrane perturbations due to production of protein adducts (Adachi et al., 2003; Worrall et al., 2001). However, prior work has also demonstrated that EtOH‐induced weakness occurred independent of peripheral nerve stimulation, in which, extensor digitorum longus muscle from mice fed EtOH produced less force than controls when using muscle‐stimulated ex vivo physiology (Crowell et al., 2019). Taken together, it would appear that neuromuscular excitation, subsequent to the central nervous system, remains functionally intact after chronic EtOH consumption and is not contributing to weakness.

### Muscle quality

Reduced muscle quality was defined as a decrease in the muscle's intrinsic force‐producing capacity and attributed to excitation–contraction coupling failure, impaired cross‐bridge cycling, and/or altered muscle composition (e.g., increased fibrosis). By normalizing muscle function to lean mass or muscle wet mass (i.e., specific force), we were able to measure how much force was produced per unit of lean mass or muscle mass. Here, we did not observe significant changes in specific force as measured by grip strength normalized to lean mass or plantarflexor torque and dorsiflexor torque normalized to posterior crural muscle mass and anterior crural muscle mass, respectively (Figure 6). In addition to specific force measures being similar in EtOH and control mice, we also did not observe a reduction in the twitch‐to‐tetanic torque. These results are similar to reports that observed ex vivo or in situ specific force did not change after mice consumed high concentrations of EtOH for at least 14 weeks (Berk et al., 1975; Crowell et al., 2019). Furthermore, Crowell et al. (2019) demonstrated the ex vivo twitch‐tetanic force ratio assessed in extensor digitorum longus muscle was similar, regardless of treatment (chronic EtOH intake vs. no EtOH) (Crowell et al., 2019). These results suggest that muscle quality assessed using various functional outcomes is not impaired after excessive, long‐term EtOH consumption.

Although the present study did not observe changes in muscle quality measured by specific forces (Figure 6) or the twitch‐to‐tetanic torque ratio, histological and biochemical studies in humans and rodents have observed that indices of muscle quality are influenced by EtOH. For instance, several reports have noted various markers of fibrosis increased in skeletal muscle after chronic EtOH consumption (Dekeyser et al., 2013; Steiner et al., 2015; Wang et al., 2012), which, if severe enough, could impact reduced specific force. Another example is the sarcoplasmic‐endoplasmic reticulum Ca^2+^ ATPase (SERCA) (Ohlendieck et al., 2003), a protein involved in Ca^2+^ homeostasis and required for excitation–contraction coupling to occur. Specifically, SERCA protein content and activity were increased after rats consumed EtOH for 6 weeks, to which the authors suggest may be an adaptive mechanism rather than a detrimental response (Ohlendieck et al., 2003). As will be discussed in the Future Directions section, long‐term adaptations may occur to overcome EtOH‐induced damage (i.e., tolerance) in order to maintain muscle function. Indeed, based on specific forces, it appears muscle quality is not impaired functionally despite others observing histological and biochemical changes (Adachi et al., 2003; Dekeyser et al., 2013; Ohlendieck et al., 2003; Steiner et al., 2015; Wang et al., 2012; Worrall et al., 2001).

### Muscle quantity

Atrophy is probably the most well‐characterized consequence of alcoholic myopathy. In fact, erosion of skeletal muscle mass has been reported to be up to 20% in humans and rodents after excessive, long‐duration EtOH intake (Crowell et al., 2019; Estruch et al., 1998; Lang et al., 2005; Martin et al., 1985; Urbano‐Márquez & Fernández‐Solà, 2004). Here, we observed that chronic EtOH consumption for approximately 40 weeks reduced lean mass and muscle wet mass of the hindlimb muscles by 9% and 14%, respectively (Figure 5). Moreover, measures of muscle atrophy paralleled force deficits, whether that was weakness observed for voluntary grip strength or in vivo electrically‐stimulated torques (Figure 2). In concert with these findings, using ex vivo mouse muscle physiology, Crowell et al. (2019) also concluded loss of muscle quantity (reductions in muscle mass and CSA) as the primary mechanism for weakness after chronic EtOH consumption.

Muscle size or quantity is dictated by the interplay between protein degradation and protein synthesis. It is currently thought that chronic EtOH consumption reduces activity of mammalian target of rapamycin complex 1 (mTORC1), a key regulator of protein synthesis (Kimball & Lang, 2018; Lang et al., 1999; Souza‐Smith et al., 2016). As a result, several downstream protein targets governing translation initiation and elongation are hypophosphorylated, impairing synthesis of both sarcoplasmic and myofibrillar proteins (Kimball & Lang, 2018; Lang et al., 1999; Reilly et al., 2000; Souza‐Smith et al., 2016). Nebulin (Hunter et al., 2003; Shenkman et al., 2019), titin (Hunter et al., 2003; Shenkman et al., 2019), desmin (Reilly et al., 2000), troponin‐T (Crowell et al., 2019), troponin‐I (Reilly et al., 2000), actin (Reilly et al., 2000), and myosin heavy chain protein (Crowell et al., 2019; Hunter et al., 2003; Reilly et al., 2000) are a few of the proteins that have been reported to be preferentially reduced in skeletal muscle after long‐term EtOH consumption. Loss of these proteins has been causally linked to EtOH‐associated reductions in protein synthesis (Kimball & Lang, 2018), which would subsequently result in skeletal muscle atrophy and weakness.

### Additional considerations

#### Mouse motivation

As outlined in the Neuromuscular Excitation section, we suggest reductions in central neural drive could be partially responsible for EtOH‐induced weakness. It is also possible that there may be a minor motivational component of weakness due to EtOH. The grip strength test was completed early in the morning (9:00 a.m.) when EtOH consuming mice likely still would have elevated BACs. Although the BACs would have been much lower than the values obtained between 5:00 and 7:00 a.m. (Figure 1), we cannot conclude how chronic EtOH consumption influenced mouse motivation in the present study.

#### Caloric intake

It should be noted that an established rodent model of chronic alcohol consumption that involved mice consuming 20% EtOH in drinking water while eating chow ad libitum was followed (Dekeyser et al., 2013; Song et al., 2002; Wagner et al., 2012). Thus, mice were not pair‐fed, nor did we measure food consumption; both of which could be considered limitations in our study design. It could therefore be argued that the changes we observed in the EtOH group may have been attributed to lower caloric intake relative to that of the controls. However, even when pair‐fed feeding regimens are implemented in mouse models of chronic EtOH intake, EtOH consuming mice still weigh less than controls and possess significantly less body fat. Crowell et al. (2016) demonstrated that after C57BL/6 male mice consumed up to a 30% liquid EtOH diet for 24 weeks (21 to 25 g of EtOH per kg of body mass per day), body mass and fat mass were approximately ~10% and 35% lower, respectively, than pair‐fed control mice. These results parallel those reported in the present study when food was provided ad libitum (Figure 4). Chronic EtOH consumption has been reported to reduce white adipose tissue and adipocyte size in rodents (Crowell et al., 2016; Kang et al., 2007; Pravdova et al., 2009; Zhang et al., 2015; Zhao et al., 2015), which would explain the similarities between our ad libitum feeding and the pair‐fed feeding of Crowell et al. (2016).

It is also important to consider that the extra weight in the control mice was largely attributed to increased fat mass. Stated differently, even if the control group (vs. the EtOH group) consumed more calories during adulthood, those extra calories would not necessarily translate into muscle hypertrophy but rather increased adiposity. Inclusion of food intake would indeed add value to alcohol‐related studies that use ad libitum feeding regimens, but based on the aforementioned comments, the numerous experiments that demonstrate histological and biochemical changes to skeletal muscle after chronic EtOH intake (Adachi et al., 2003; Fernandez‐Solà et al., 2007; Kimball & Lang, 2018; Simon et al., 2017), and the ex vivo muscle physiology and body composition data published by Crowell et al. (2016, 2019), we suggest that our results were primarily due to EtOH not calorie intake (if it did indeed differ between groups).

#### Aging and development

We would like to emphasize that it is possible that chronic EtOH consumption in the present study (and others; Crowell et al., 2019) may have hindered mouse development. Theoretically, the weakness observed in the EtOH group may have been due to impaired developmental hypertrophy rather than increased atrophy. In attempt to limit this possibility, EtOH feeding began at 23 weeks of age. However, without testing mice over the entire duration of the study to specifically document that EtOH reduced grip strength, in vivo muscle contractility, and lean mass from peak baseline values, we cannot completely rule out this scenario. As will be mentioned in following paragraph, longitudinal research designs will be necessary to advance EtOH‐related research.

#### Future directions

Although this was the first comprehensive in vivo study to establish chronic EtOH consumption results in skeletal muscle weakness in female mice and identify possible neuromuscular mechanisms for this weakness, we would like to highlight how future physiological studies can improve on these findings. First, we specifically used fast‐twitch muscle of female mice because human and rodent data suggest females (Nemirovskaya et al., 2015; Shenkman et al., 2019; Slavin et al., 1983) and fast‐twitch muscles (Kimball & Lang, 2018; Lang et al., 1999; Preedy et al., 2003; Slavin et al., 1983) are more sensitive to developing alcoholic myopathy. This however does not mean males or slow‐twitch muscles need not be assessed, but rather used in conjunction to females and fast‐twitch muscle in order to identify mechanisms of EtOH‐related resiliency. Second, in the present study we only assessed muscular force and neuromuscular mechanisms of weakness after long duration, chronic consumption. It is possible we missed important changes that occurred prior to significant reductions in muscle quantity and/or neuromuscular aspects of EtOH‐induced tolerance. Skeletal muscle is extremely plastic, and it would therefore be an oversight to assume the muscle would not “adapt” to damage imposed on it by EtOH. Future studies will also need to be longitudinal in nature and assess neuromuscular function as alcoholic myopathy develops and progresses. Lastly, we would like to emphasize that we only measured function under short length contractions without any physiological stress, such as conditions of fatigue or injury. It is possible that neuromuscular mechanisms of weakness after chronic EtOH consumption may be different under stressed vs. basal conditions.

## CONCLUSIONS

To determine if chronic EtOH intake causes weakness in mouse skeletal muscle and if so, the underlying neuromuscular mechanisms responsible, we measured in vivo strength and contractility, while also assaying several indices of neuromuscular excitation, muscle quantity, and muscle quality. Here, we are the first to report EtOH reduces the muscle's force producing capacity in vivo after chronic consumption in mice. Moreover, we provide evidence that the primary neuromuscular mechanism causing weakness was muscle atrophy, while reductions in central neural drive may also have contributed, albeit to a much smaller degree. Future investigations are required to understand the pathophysiology of EtOH‐induced damage as it relates to the development and progression of weakness, as the present study assessed a single timepoint after weakness was already apparent.

## FUNDING INFORMATION

This work was funded, in part, by a grant from the NIH/NIA (R01AG067758).

## CONFLICT OF INTEREST

In the past 5 years, Brian Clark has received research funding from NMD Pharma, Regeneron Pharmaceuticals, Astellas Pharma Global Development, Inc., and RTI Health Solutions for contracted studies that involved aging and muscle‐related research. In the past 5 years, Brian Clark has received consulting fees from Regeneron Pharmaceuticals, Zev industries, and the Gerson Lehrman Group for consultation specific to age‐related muscle weakness. Brian Clark is a cofounder with equity of OsteoDx Inc. In the past 5 years, W. David Arnold has received research funding from Biogen, Novartis, Genentech, NMD Pharma, and Avidity Biosciences.
